# Role of Exopolysaccharide in *Aggregatibacter actinomycetemcomitans*–Induced Bone Resorption in a Rat Model for Periodontal Disease

**DOI:** 10.1371/journal.pone.0117487

**Published:** 2015-02-23

**Authors:** Mayilvahanan Shanmugam, Prerna Gopal, Faiha El Abbar, Helen C. Schreiner, Jeffrey B. Kaplan, Daniel H. Fine, Narayanan Ramasubbu

**Affiliations:** Department of Oral Biology, Rutgers School of Dental Medicine, Newark, NJ, 07103, United States of America; Columbia University, UNITED STATES

## Abstract

*Aggregatibacter actinomycetemcomitans* a causative agent of periodontal disease in humans, forms biofilm on biotic and abiotic surfaces. *A*. *actinomycetemcomitans* biofilm is heterogeneous in nature and is composed of proteins, extracellular DNA and exopolysaccharide. To explore the role played by the exopolysaccharide in the colonization and disease progression, we employed genetic reduction approach using our rat model of *A*. *actinomycetemcomitans*-induced periodontitis. To this end, a genetically modified strain of *A*. *actinomycetemcomitans* lacking the *pga* operon was compared with the wild-type strain in the rat infection model. The parent and mutant strains were primarily evaluated for bone resorption and disease. Our study showed that colonization, bone resorption/disease and antibody response were all elevated in the wild-type fed rats. The bone resorption/disease caused by the *pga* mutant strain, lacking the exopolysaccharide, was significantly less (P < 0.05) than the bone resorption/disease caused by the wild-type strain. Further analysis of the expression levels of selected virulence genes through RT-PCR showed that the decrease in colonization, bone resorption and antibody titer in the absence of the exopolysaccharide might be due to attenuated levels of colonization genes, *flp-1*, *apiA* and *aae* in the mutant strain. This study demonstrates that the effect exerted by the exopolysaccharide in *A*. *actinomycetemcomitans*-induced bone resorption has hitherto not been recognized and underscores the role played by the exopolysaccharide in *A*. *actinomycetemcomitans*-induced disease.

## Introduction

It has been well documented that biofilm bacteria predominate, numerically and metabolically, in virtually all nutrient-sufficient ecosystems, including the oral cavity [1,2]. Bacterial cells in biofilms are surrounded by a self-synthesized, three-dimensional matrix (slime or extracellular polymeric substance), which holds the cells together and firmly attaches the bacterial cells to the underlying surface [3]. The extracellular polymeric substance has been attributed to a protective role as well as it is a source of dissolved nutrients, secreted enzymes, extracellular DNA and exopolysaccharide. The exopolysaccharide of *Aggregatibacter actinomycetemcomitans* (PGA) is a homopolymer of N-acetyl-D-glucosamine residues in β(1,6) linkage and has been well characterized in several bacteria including *Staphylococcus aureus*, *S*. *epidermidis* and *E*. *coli*. This exopolysaccharide has been named differently in various bacteria but its synthesis is encoded by a set of four genes, *icaADBC* in Staphylococcal species and *pgaABCD* in *E*. *coli* and *A*. *actinomycetemcomitans*. The exopolysaccharide from *A*. *actinomycetemcomitans*, PGA, is a surface-associated polymer that can protect *A*. *actinomycetemcomitans* at the cellular level from phagocytic killing [4]. A similar protective function was ascribed to the exopolysaccharide PIA of *S*. *epidermidis* [5]. PGA/PIA mediates resistance to killing by antibiotics [6], detergents [7] and antimicrobial peptides [5]. PGA may act through a general mechanism wherein it binds to or electrostatically repulses immune modulators and antimicrobial agents, thereby preventing their access to the bacterial cell [5].

In this report, we have focused our attention on the oral bacterium *A*. *actinomycetemcomitans* that is a causative agent of localized aggressive periodontitis (LAP). In this disease state ligamentous tissue and alveolar bone surrounding first molars and central incisors are lost rapidly resulting in eventual tooth loss. In particular, the incidence of occurrence of this disease is 15 times more prevalent in African-American adolescents compared to the general population [8]. Recently, accumulating evidence has suggested that *A*. *actinomycetemcomitans* is required to initiate LAP, which occurs with a higher frequency not only in children of African American descent (2%) but also Hispanic (1%), Caucasian (0.1%) and Asian descent [9,10]. Even though it is clear that chronic adult periodontitis is a more significant public health issue when compared to LAP, unraveling the initial steps in *A*. *actinomycetemcomitans*-induced LAP should be applicable to microbes involved in chronic adult periodontitis as well, since similar adaptive mechanisms are required [8,11,12]. Our focus is to study the central role played by the PGA in the infectious disease process of *A*. *actinomycetemcomitans* (attachment, colonization, persistence).

Animal models have been used to study bacterial pathogenesis and to establish a role for a particular gene product in the process. Generally, the involvement of a gene product is ascertained by comparing the outcome provoked by the wild-type strain and a genetically modified strain. To study the disease process caused by *A*. *actinomycetemcomitans*, such a model was developed, which showed that rats fed with wild-type strain (IDH781) produced significant bone resorption compared to the non-fed control group [13]. When the involvement of specific genes such as *tadA* or *flp*-1 was tested, it was concluded that these were required for colonization [13]. In another study testing *ltxA* or *cdtB* mutant strains, the bone resorption induced by the *ltxA* mutant strain was significantly less than that of the wild-type strain. In contrast, the bone resorption induced by the *cdtB* mutant strain was comparable to the wild-type strain [14]. While pathogenicity of *A*. *actinomycetemcomitans* has been associated with many virulent genes including *ltxA*, *flp*-1, *tadA*, *luxS* and *cdtB*, how PGA production modulates the expression of these genes is not yet understood.

The importance of PGA in a protective role for *A*. *actinomycetemcomitans* and other bacteria has been well established [4]. In addition, a recent study of Ps1, the exopolysaccharide of *P*. *aeruginosa*, has demonstrated that it also determines the fate of elite cells in the initial microcolony development [15]. While these studies highlight the significance of exopolysaccharide, they also bring the genes encoding the exopolysaccharide to the forefront in the disease process. For example, in several infection models, the exopolysaccharide PIA has been demonstrated to be relevant for the virulence of *S*. *epidermidis* [16,17,18]. These studies highlighted the contribution of PIA to the chronic and persistent nature of *S*. *epidermidis* biofilm infections. Therefore, we began to explore the contribution of PGA for *A*. *actinomycetemcomitans* virulence using the redefined rat model of periodontitis and compare the bone resorption to a genetically modified strain, which lacks the entire *pga* operon (*pga* deletion mutant, EA1002; [19]). This phenotype is a rough phenotype based on the morphology when grown on agar plates. Our hypothesis is that production of PGA is necessary for *A*. *actinomycetemcomitans* to induce bone resorption and that lack of PGA production will modulate important virulence genes likely due to physiological changes. In this study, we show that in the absence of PGA synthesis, the *pga*-lacking strain (EA1002; [19]) does not cause bone resorption, which is comparable to the no-bacteria fed control group of rats. Further, we also report on the changes in the gene expression levels of virulence genes in the early stages of growth to understand the initial colonization process.

## Materials and Methods

### Bacterial strains, media, and growth conditions

*A*. *actinomycetemcomitans* strain IDH781, serotype d, a clinical isolate [20] and EA1002 strain [19] were grown on TSA plates containing yeast extract (0.6%), sodium bicarbonate (0.4%), glucose (0.75%) [21] and 30 μg/mL rifampicin for 2 days in a 37°C incubator at 10% CO_2_/90% air atmosphere. The cells were scraped from the agar plates, suspended in PBS, washed twice with PBS and suspended in PBS containing 3% sucrose. Suspended cells were mixed well by vortexing for 30 sec and adjusted to ∼10^8^ cells per mL (OD_600_ = 0.80).

### Confocal microscopy for *in vitro* biofilms

For confocal microscopy, 35 mm glass bottom microwell culture dishes (Cat. # P35G-0-10-C.s, MatTek Co. Ashland, MA) were used and 2 mL of wild-type or EA1002 cells grown as before were seeded and incubated at 37°C (10% CO_2_) for 16 h. Cells were washed with fresh prewarmed TSB (37°C) containing yeast extract (0.6%), sodium bicarbonate (0.4%) and glucose (0.75%) [21] and 2 mL fresh medium was added. Cells were stained with Film tracer biofilm LIVE/DEAD (Life Technologies, NY) kit by adding the stain directly to the TSB medium in the dishes and incubated at 37°C (10% CO_2_) for 20 min. Cells were washed with fresh prewarmed medium (37°C) and the images were acquired with Nikon A1R-A1 confocal microscope using objective lens Plan Apo VC 60x WI DIC N2. The acquired images were processed and the volume of live/dead cells were measured in 7 different zones and averaged. The % of dead cells was calculated as dead/total times 100. The means of the dead cells in wild-type and EA1002 were subjected to Student’s *t*-test for significance (P < 0.05). All measurements were calculated using NIS Elements software (Version 4.2).

### Inoculation of Rats

**Fig 1:**
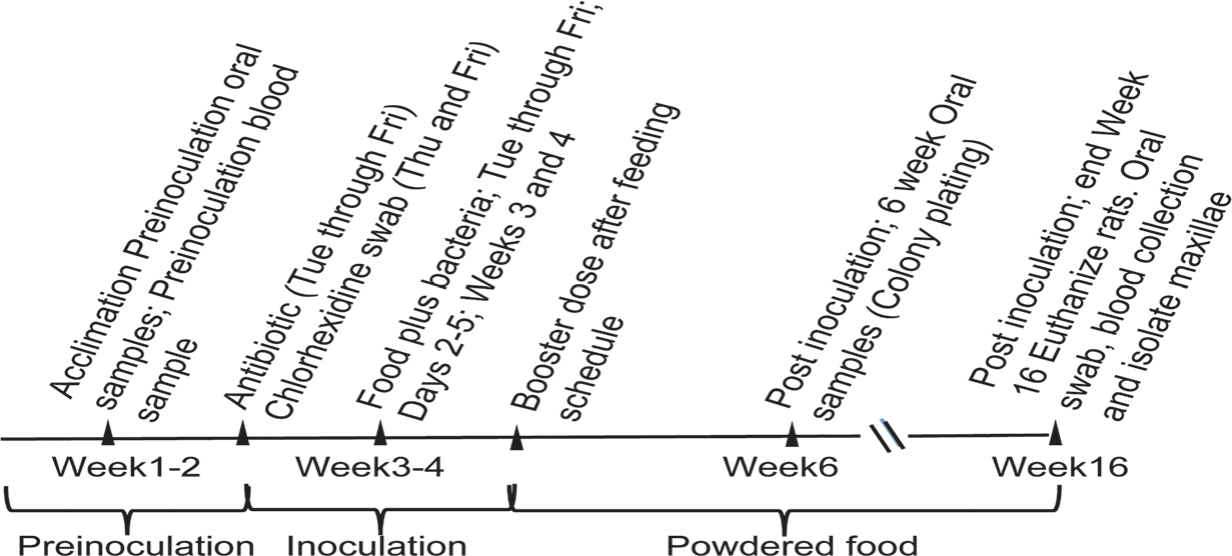
Scheme for colonization and bone resorption.

This study was carried out in strict accordance with the recommendations in the Guide for the Care and Use of Laboratory Animals of the National Institutes of Health. The protocol was approved by the Institutional Animal Care and Use Committee at Rutgers (Animal Care and Use Protocol #12073C0915). All efforts were made to minimize suffering of the rats during the length of the experiment. This procedure was essentially the same as reported earlier except that a booster dose of bacteria was fed to the rats [13]. The scheme for time line for colonization and bone resorption is given in Fig. 1. Three groups of six pathogen-free, Sprague–Dawley male rats, 6–8 weeks old and weighing 150–250 g (Taconic, Germantown, NY), were used in the study. Group I was fed wild-type strain IDH781 Rif, Group II was fed a *pga* mutant, EA1002 Rif [19], Group III served as the non-infected control. The rats were housed in separate cages and fed Laboratory Rodent Meal Diet 5001 (Purina Mills Feeds, St. Louis) [22]. Naturally occurring resident flora were suppressed by the use of antibiotic (20 bone resorption each of ampicillin and kanamycin) in the water provided to the rats for a period of 4 days [23]. The mouths of the rats were swabbed with 0.12% chlorhexidine gluconate rinse (Peridex, Procter and Gamble, Cincinnati) during the last 2 days of antibiotic treatment. The rats were unfed for 3 h prior to the feeding with either bacteria or control mix; the inoculum consisted of 10^8^ cells in the food while the food mix for the control rats were without any bacteria. For feeding, one gram of powdered food was mixed with 1 mL of PBS containing 3% sucrose and 1 X 10^8^ respective bacterial cells on 60 mm cell culture plate and placed inside of the cage. The food was left in the cage for 1h and then removed. After 8 days of feeding, a booster dose of bacteria was given to infected rats (EA1002 and wild-type fed) to ensure colonization of the bacterial strains. Booster dose was prepared by collecting respective bacterial cells from the agar plates, grown for 48 h in 10% CO_2_, and suspended in PBS (pH 7.4) to make a thick slurry. The cells were taken up on a cotton swab, presoaked in PBS and applied to the teeth, palate, cheek and the tongue of the rats. The rats were anaesthetized and held such that there was minimal to no movement of the heads. This treatment was undertaken to ensure that sufficient bacteria were present to initiate colonization in both EA1002 and wild-type fed rats. For the control group, we applied cotton swabs soaked in buffer to the mucosa.

### Sampling of the oral flora

As in the previous studies, oral flora was sampled after 2 and 12 weeks post inoculation. The samples were treated as described before [13]. Selection of individual colonies of *A*. *actinomycetemcomitans* was performed on TSA plates containing 30 μg/mL Rif using 10-fold and 100-fold dilutions of the swab samples. Plates were incubated at 37°C for 5–7 days (total bacteria) or in the presence of 10% CO_2_ at 37°C for 2–3 days (*A*. *actinomycetemcomitans* selection). When necessary, PCR amplification of the *ltxA* gene was performed to verify the presence of *A*. *actinomycetemcomitans* strains. For this, the genomic DNA from each strain was isolated by using Qiagen kit (Qiagen, Hilden, Germany) and leukotoxin specific forward primer 5’ AGG TAT TGC GAA ACA AT 3’ and reverse primer 5’ GAA ATT AAG CTG GTA AT 3’ were used.

### Assay for bone resorption

**Fig 2:**
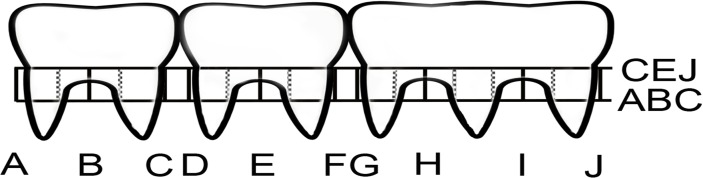
Sites for *A*. *actinomycetemcomitans*-induced bone-loss measurement. Three molars of one side of the rat maxillae are shown. Sites A through J were numbered similarly in side of the jaw and measurements were taken from the CEJ to the ABC. Dashed lines indicate where additional measurements were made as and when necessary.

After the 12-week post-inculation sampling for plaque samples, sodium pentobarbital (100 mg/kg i.p.) was used to kill the rats. The heads were removed and stored at -70°C when necessary. Rat maxillae were cleaned and defleshed and digital photography methods that measures bone resorption from the cemento-enamel junction (CEJ) to the alveolar bone crust (ABC) using a methylene blue stain and visual assessments were used [23,24]. This method has been shown to provide reliable bone resorption data over two other methods used previously [24]. The maxillae were stained with 1% methylene blue, and digital images were recorded with a DP12 microscope digital camera (Olympus, Center Valley, PA, USA) at a magnification of 9.2. The jaws were positioned with the lingual side facing up so that the occlusal plane of the molars was perpendicular to the microscope stage. The photos were printed on an HP Color printer on 4x6 glossy paper (Kodak Ultima Picture Paper, Eastman Kodak Co.) and coded so the examiners were unaware of the treatment groups. The vertical distance between the CEJ and the ABC were measured at 10 sites (sites A, B, C, D, E, F, G, H, I and J) on both right and left maxillary jaws (Fig. 2). For improved precision, the distance was measured by using an electronic digital caliper (Marathon; Control Company, Friendswood, TX, Model- 14-648-17). When necessary, each tooth was divided further into additional sites and the measurements for that tooth were averaged.

### Analysis of antibody against *A*. *actinomycetemcomitans* strains

Initial bleeds were obtained from the tail vein before bacterial feeding and a final bleed by cardiac puncture. Serum samples were stored at -70°C. *A*. *actinomycetemcomitans* lysates were prepared from 2-day cultures (adjusted to 10^8^ bacteria/mL; OD_600_ = 0.8), re-suspended in PBS and TEN buffer [13]. Background levels of antibody that cross-reacted with *A*. *actinomycetemcomitans* were determined by comparison to control rat sera and preimmune sera. All assays were run in triplicate. The enzyme-linked immunosorbent assay (ELISA) was used to determine IgG antibody to *A*. *actinomycetemcomitans* as described previously [13,25].

Briefly, the nunc-immuno micro titer plates (Sigma-Aldrich, St. Louis, MO) were coated with serially diluted 100 μL of *A*. *actinomycetemcomitans* strain (wild-type as well as EA1002) lysates or rabbit IgG antibody (10 μg/mL) and incubated overnight. A separate experiment was carried out to obtain the standard curve. The plates were washed with TBS (20 mM Tris buffered saline, pH 7.5) and blocked with blocking buffer for 1h at room temperature (3% BSA in TBS). The plates were washed three times with TBS and the diluted rat serum samples (1% BSA in TBS, 1:250), including the prebleed samples were added to the wild-type or EA1002 lysate coated wells and incubated at room temperature for 2 h. Then the plates were washed with TBS containing 0.05% Tween-20 (x4). The anti-rat IgG Fc-specific alkaline phosphatase conjugated secondary antibody was diluted (1:30,000; 1% BSA in TBS; Sigma-Aldrich, Catalog No. SAB3700542) and added to the wells and incubated for 1 h at room temperature. The wells were washed with TBS-Tween-20 (0.05%) five times after the secondary antibody incubation. The wells were finally washed with TBS alone. The enzyme substrate was prepared by dissolving the tablets of *p*-nitrophenyl phosphate in 10% diethanolamine buffer and 100 μL was added to the wells. The plates were incubated for 30 min at room temperature and absorbance of the end product, *p*-nitrophenol, was measured at 405 nm using a Synergy H1 hybrid multi-mode micro plate reader (BIO-TEK, Winooski, VT). The data were collected using the Gen5 analysis software.

### Statistical analysis

Based on our previous studies, a sample size was fixed at six rats/group since this number was adequate to detect statistically significant differences between groups with 80% power [13]. The total area measured from each rat in each group was used to determine the amount of bone resorption for each of three groups studied. One-way ANOVA and Scheffe post-hoc analysis (Stata/MP 13.1) were performed for comparing the total bone resorption among the various treatment groups. The average total bone resorption of the six rats in each group was considered statistically different if the *P* value was < 0.05. Similarly, we also tested if statistically different amounts of bone resorption per site occurred in the rats fed the wild-type or EA1002 mutant strains as compared with the control rats that were not fed *A*. *actinomycetemcomitans* [24]. Elevated antibody titers and colonization were analyzed by testing whether or not these values were above background seen in control group of rats. This data was evaluated by a χ^2^ analysis and to achieve significance a *P* value of 0.05 was required. Also, as described before [24], the average of the bone resorption per site in control group was used to test whether or not a particular site in an infected rat exhibited true bone resorption. For this test to be true, the site being examined was required to show a level of bone resorption that was greater than two standard deviations above the mean bone resorption at that same site in the control rats. Further, for a rat to be diagnosed with ‘‘real’’ disease, that rat was required to have ‘‘true’’ bone resorption at two or more sites in any one quadrant [24]. Data from qPCR experiments and from the % of live/dead cells were analyzed by unpaired Student’s t-test for significance (GraphPad Prism 6).

### RNA isolation and purification

Wild-type and *pga* mutant strain EA1002 were grown on tryptic soy agar plates for 48 h, cells were collected by scraping, and suspended in tryptic soy broth. Cells were uniformly distributed by vigorous vortexing, and optical density was measured (OD_600_). The suspended cells were diluted to ∼ 1 X 10^5^/mL and were plated on 10 cm plastic plates for biofilm growth by incubating at 37°C with 10% CO_2_ for 16 h. The biofilm growth to 16 h was chosen based on our earlier experiments [19] where we showed that the 24 h biofilm growth completely covered the plate surface. Since such a growth is considered a mature biofilm and because of our interest in finding the gene expression before the biofilm becomes mature, 16 h was chosen as the time point. The growth medium is conducive for biofilm growth and only those cells that were attached to the surface were collected. Free floating cells and the medium supernatant was removed and the biofilm RNA was stabilized by adding ice cold 0.9% saline supplemented with 1/10^th^ volume of 95% ethanol and 5% citric acid saturated phenol mixture. The attached biofilm cells were scraped, transferred with the saline to a centrifuge tube and centrifuged at 10,000 rpm for 5 min at 4°C. Pelleted cells were flash frozen in liquid nitrogen and stored at -80°C until further use.

The hot-phenol method of RNA isolation was used for wild-type as well as EA1002 biofilm cells with modifications to improve the quality of RNA. Briefly, glass beads were added to frozen pelleted biofilm cells followed by the addition of 700 μL ice cold suspension buffer (30 mM sodium acetate pH 4.3, containing 1% β-mercaptoethanol, 2 mM aluminum ammonium sulphate, 2 mM EDTA). The cell suspension was bead-beaten (Biospec Products, Bartlesville, OK) for 10 sec to make sure the cells were dispersed evenly. To the pellet, 102 μL of preheated (65°C) lysis buffer (300 mM sodium acetate pH 4.3, 10% β-mercaptoethanol, 8% SDS, 16 mM EDTA pH 8.0) was added, bead-beaten for 20 s, and incubated at 65°C for 3 min. Equal volume of preheated acidic phenol saturated with citrate buffer, pH 4.0 (65°C) was added and bead-beaten for 20 s (5X) with 1 min intervals maintaining the temperature at 65°C. The phenol mixture was centrifuged at 14,000 rpm for 20 min at 4°C. After repeating with acidic phenol extraction, the aqueous phase was extracted with ice-cold chloroform (2X). Finally the RNA was precipitated with the addition of 10% sodium acetate and 100% ethanol (2.5 volumes). The ethanol precipitated RNA was pelleted down by centrifugation at 14,000 rpm for 20 min and washed twice with 70% ethanol. The RNA pellet was air dried and suspended in DEPC treated water. Total RNA was quantitated using Nanodrop Lite spectrophotometer (Thermofisher Scientific, Wilmington, DE). The quality of the RNA was determined by the ratio of absorbance at 260/280 nm. To improve the quality of the total RNA, samples were passed through the Micro Bio-Spin P-30 Gel Columns (Bio-RAD, Cat No. 732-6250).

After gel filtration step, RNA samples were quantitated and 500 ng of total RNA was mixed with RNA sample loading buffer, denatured at 65°C for 10 min, ice cooled and analyzed on 1% TAE agarose gel. The RNA integrity was confirmed by visualizing the staining intensity difference between 23S and 16S rRNA. The purified total RNA samples were stored at -80°C until further use. The total RNA (5 μg) was subjected to DNase I treatment to remove the contaminant genomic DNA using the RNA purification Kit (Zymo Research, Irvine). As negative control, complete DNA removal was confirmed in all the samples by PCR using *apiA* primers (Table 1) before subjecting the samples for qPCR.

**Table 1:** Primers used for qPCR analysis.

Gene	Forward Primer	Reverse Primer
*ltxA*	ATCAGCCCTTTGTCTTTCCTAG	TGACCAAGTAAACTATCGCCG
*aae*	TGGTGCTACTTCTGTTTCCTC	ATCTTGAACCTAGTGTGGCTG
*cdtB*	TGCATTAAGACAGGAACCCG	TGCTCTCGACGTGGTAAATG
*apiA*	CTCTACATCAGCCTTATCCGC	TGATGGAAATCTGGAAGGCG
*flp*	TCAAAGCAGCTGAAGCAATC	GCGATAAAACCATTGTTGCTG
*tadA*	TGGCAGCCGTTTAAATGTTG	ATTTCGCGAGTCATAGAACC
*katA*	AATTCAAATCATGCCGGAAG	TGCGATTCAGTTCCAATACG
*16S rRNA*	AAATGCGTAGAGATGTGGAGG	TATCTAATCCTGTTTGCTCCCC

### Quantitative PCR

For the cDNA synthesis, high capacity cDNA synthesis kit from Life Technologies (Grand Island, NY) was used following the manufacturer’s instructions. After the cDNA synthesis, part of cDNA was diluted to 1:25 for mRNA amplification and 1:7000 for 16S rRNA amplification. For quantitative PCR, the Roche SYBR green master mix was used to amplify the gene targets using LightCycler 480 system and the data were analyzed using LightCycler 480 software (Version 1.2.9.11), using 16S rRNA as internal control using the primers listed in Table 1. Using the appropriate primers, the expression of the various genes was assessed in the two strains. The fold change in the gene expression levels between the wild-type and the EA1002 strains was calculated based on 2^−ΔΔCт^ value compared to 16S rRNA.

## Results

### Confocal microscopy: Effect of *pga* operon and biofilm architecture

**Fig 3:**
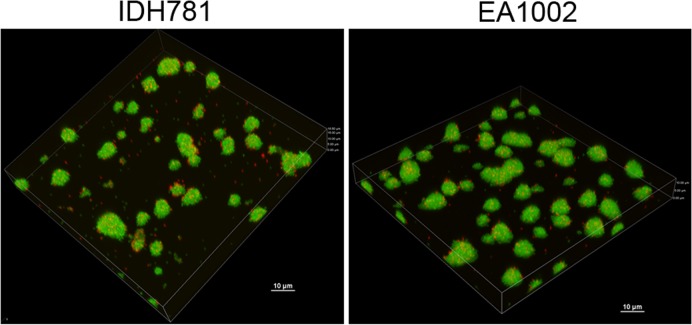
Confocal scanning laser microscopic image of biofilms. Biofilm growth was imaged at seven different locations and averaged for analysis.

Biofilms were grown for the EA1002 mutant strain and the wild-type strain and compared using confocal microscopy using Live/Dead Filmtracer fluorescent stain (Life Technologies, Grand Island, NY). The 16 h time period for biofilm growth was chosen throughout this study to focus on the initial colonization events wherein many of the virulence genes would be active. When analyzed by confocal microscopy, the biofilm colonies were no different from the colonies from the wild-type strain. Both strains exhibited the typical 3D biofilm structure characteristic of multicellular communities (Fig. 3). However, the amount of dead cells in the EA1002 biofilm, measured as the % volume of the total biofilm, was significantly less than the amount in the wild-type (6.65±0.60% vs. 10.06±1.57%, respectively) with a *P* value of 0.0002. Clearly, more of the wild-type cells were dead compared to PGA-lacking cells at 16 h. In contrast, at 48 h, there is more cell death in the EA1002 strain compared to the wild type strain biofilm (Wild-type 72±1% vs. EA1002 99±1%; P <0.0001). When grown in broth cultures, the mutant strain exhibited no apparent defect in the growth [19].

### Effect of *pga* operon on periodontal disease: Antibody response

The effect of PGA on the colonization and *A*. *actinomycetemcomitans*-induced bone resorption was determined using the rat model developed previously. As mentioned in the Methods, three groups of rats were included in the study: a) uninoculated control group; b) EA1002 fed group and c) the wild-type fed group. The antibody titer for the three groups in this study representing the immune response was examined using ELISA with sera collected 12-week post-infection. The lysate from the bacterial strain used for feeding the rats was reacted against the antiserum from the individual rats. The mean antibody equivalence levels (OD_405_ values) are presented in Table 2. The level of antibody to *A*. *actinomycetemcomitans* was higher in both the *pga* mutant as well as the wild-type inoculated groups compared to the preimmune sera, respectively. In addition, in both the cases, the antibody levels in the inoculated strains were significantly higher than the control group suggesting that the colonization of *A*. *actinomycetemcomitans* was sustained through at least 12 weeks (post-inoculation) in the mutant as well as the wild-type fed rats (One-way ANOVA, *P* value = 0.0001).

**Table 2:** *A. actinomycetemcomitans* induced colonization, antibody level and bone resorption.

Group	Disease	Disease site	Total bone resorption (mm)	Antibody Concentration 12 weeks (post inoculation) (μg/mL)	Colonization (cfu/mL) 2 weeks
Control					
1–1	No	None	143.7	0	0.000
1–2	No	None	153.7	0	0.000
1–3	No	None	147.1	0	0.000
1–4	No	None	141.5	0	0.000
1–5	No	None	114.6	0	0.000
1–6	No	None	157.2	0	0.000
Mean ± SD			142.9 ± 15.1		
EA1002					
2–1	No	None	141.9	193	0
2–2	No	None	130.4	438	0
2–3	No	None	129.3	228	0
2–4	No	JL	144.5	119	0
2–5	No	None	138.8	244	260
2–6	No	None	134.3	364	20
Mean ± SD			136.5 ± 6.2	263 ± 116	
Wild-type					
3–1	Yes	IL,JL, GR,HR,IR^‡^	172.4	134	0
3–2	Yes	IR,JR	160.1	322	240
3–3	Yes	EL—JL; DR—JR	200.4	194	70
3–4	Yes	AL-CL, FL; AR, DR-GR,	184.0	594	70
3–5	No	JL, JR	156.5	300	350
3–6	No	JL, AR, JR	156.05	414	20
Mean ± SD			171.6 ± 17.8^†^	326 ± 124^$$^	ND

^†^ P = 0.0006, 0.0029, against control and EA1002, respectively.

‡ L = left; R = right; A-J = sites for bone resorption analysis [24].

^$$^ P = 0.741 for EA1002 vs. wild-type; P = 0.001 for wild-type vs. Control and P = 0.004 for EA1002 vs. Control.

### Colonization

**Fig 4:**
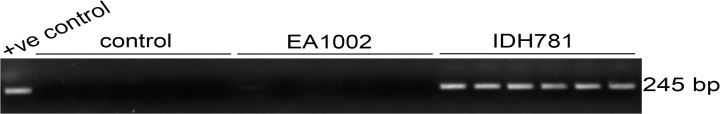
Amplification of leukotoxin gene using genomic PCR for recovery of *A*. *actinomycetemcomitans* from rats at 12-weeks post infection. The *A*. *actinomycetemcomitans* genomic DNA was used as a positive control. Only wild-type (IDH781) fed rats show the presence of *ltxA*.

The *pga* mutant clearly exhibited less colonization compared to the wild-type but comparable to the control group that was not fed any bacteria. The colonization was evident at 2 weeks for both the *pga* mutant as well as the wild-type strain fed rats. Only two of the six rats in the *pga* mutant group exhibited colonization whereas five out of six rats in the wild-type group exhibited colonization (Table 2). However, *A*. *actinomycetemcomitans* CFU could not be recovered after 12 weeks (post-inoculation) in all the groups. To test whether the failure to recover *A*. *actinomycetemcomitans* from the inoculated rats is due to sampling or the result of reduced bacterial recovery, we performed PCR to test the presence of the *A*. *actinomycetemcomitans* strains. We selected *ltxA* as the gene for this analysis and as seen in Fig. 4, only the wild-type fed rats exhibited the amplification of *ltxA* suggesting that other factors might have affected the recovery.

### Total bone resorption and treatment group

The total bone resorption was significantly higher in the wild-type fed rat group compared to the total bone resorption for the *pga* mutant fed group or the control group (Table 2). The total bone resorption for the control group and the *pga* mutant fed groups was comparable. The differences of the means between them were not significant (P = 0.43; One-way ANOVA). However, the differences between means of the wild-type and the other two groups, control and EA1002, were significant (P = 0.0006 and P = 0.0029, respectively). This result suggests that the three groups differ in the susceptibility to *A*. *actinomycetemcomitans*-induced bone resorption and that the bone resorption in the wild-type group is significantly higher.

We also assigned disease status to each rat in the various groups. As shown in Table 2, none of the rats in neither the control group nor the EA1002 fed group could be classified as diseased. However, 4 out of 6 rats turned out to be diseased in the wild-type fed group. Fischer exact tests were used to compare the number of diseased rats in the control groups versus *A*. *actinomycetemcomitans*-inoculated groups for each rat strain (P = 0.001). We also looked at the bone resorption at the individual sites, A through J with left (L) and right (R) quadrants, as described previously [24]. We noted that the number of disease sites per rat was higher in the wild-type fed group compared to either EA1002 mutant group or the control group. The frequency of occurrence was also significantly different between the wild-type and the control or the wild-type and the EA1002 mutant group (Table 2). While none of the sites in control groups had any disease, only one site in one rat (2–4) in the EA1002 inoculated group showed bone resorption. However, in the wild-type inoculated group, all the rats had at least one site that could be classified as diseased. The wild-type fed rats had a total of 32 diseased sites, 15 on the left and 17 on the right, suggesting that there may not be a preference for any given side. Based on the bone resorption results, we conclude that lack of *pga* genes is more likely to attenuate the ability of *A*. *actinomycetemcomitans* to induce bone resorption.

### qPCR and fold change in virulence genes

**Fig 5:**
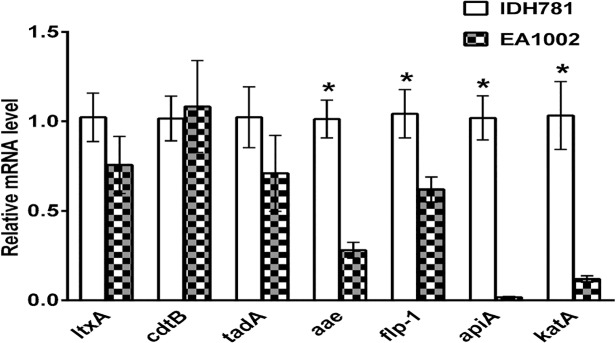
Expression of virulence genes in the *A*. *actinomycetemcomitans* strains. Total mRNA was isolated, and relative levels of the genes were quantified by qPCR as described in the text. Results are means ± standard deviations for triplicate cultures normalized to the result with 16S rRNA. The fold changes in the mRNA levels of the genes that are significantly different between the strains are shown by asterisks as measured by Student’s *t* test.

To determine whether or not the virulence gene expression is modulated by the lack of *pga* genes, we measured the expression levels of mRNA for several genes. These genes represent the well-studied colonization genes (*aae*, *apiA*, *flp-1*, *tadA*) and genes involved in immune evasion (*ltxA*, *cdtB and katA*). Total mRNA was isolated, transcribed into cDNA, and subjected to qPCR. The results shown in Fig. 5 demonstrate that the colonization related genes are affected by the absence of PGA. The fold change in the mRNA levels between the wild-type and the EA1002 strains in genes *aae*, *apiA*, *flp-1* are significant (P = 0.0004, P = 0.0001, P = 0.0085, and P = 0.001, respectively) when analyzed using Student’s t test; however, the levels of mRNA for *tadA* in the two strains were not significantly different (P = 0.1163). Interestingly, while the immune evasion genes, *ltxA*, *cdtB* were also not significantly different (P = 0.0921, P = 0.707, respectively), the fold change for *katA* gene was significant between the two strains (P = 0.0012).

## Discussion

Many pathogenic bacteria cause disease by their ability to form biofilms. The ability of *A*. *actinomycetemcomitans* to adhere tenaciously forming a strong biofilm is an important factor in the etiology of the periodontal disease [26]. There are distinct stages in biofilm formation undertaken by the pathogenic bacteria including *A*. *actinomycetemcomitans*. Among these stages, initial attachment to surfaces is important. In *A*. *actinomycetemcomitans*, initial attachment to biotic/abiotic surfaces is mediated by fimbriae [27]. In addition, other outer membrane adhesins such as ApiA and Aae, are both required for proper binding of *A*. *actinomycetemcomitans* to human buccal epithelial cells [28]. An earlier study has shown that although all the *pga* genes are required for the PGA production, the mutants lacking *pga* genes were able to form biofilms [19]. In uropathogenic *E*. *coli*, loss of PGA did not affect the ability of the modified organism to form biofilm *in vitro*. It has been previously reported that a *pgaC* mutant strain also formed biofilm and bound to polystyrene plates with the same avidity as the wild-type strain [29]. A similar observation was made in the study of exopolysaccharide mutant of *Bordetella pertussis* [30]. Taken together these studies suggest that PGA may have additional role in the *A*. *actinomycetemcomitans* biofilm growth.

We analyzed the biofilm characteristics of both the wild-type and the EA1002 strains after 16 h since characteristics of the biofilm formed by any *pga* mutant, including EA1002, hitherto has not been reported. As shown in Fig. 3, the absence of the *pgaABCD* locus does not significantly affect the early stage of biofilm formation. What is significant about the biofilm of EA1002 compared to the biofilm of wild-type is the finding that although there is more cell death in the wild-type biofilm in the early stage (16 h), at 48 h, the amount of cell death in the EA1002 strain is significantly higher. Cell death in a biofilm is a highly regulated process, which releases extracellular DNA in to the matrix [31]. During maturation of the biofilm, new bacterial cells could use the extracellular DNA for adhering to the matrix. However, the difference in the amount of cell death in two strains, wild-type and EA1002, in the early stage as well as in the mature stage, could be critical in disease pathogenesis.

Since the EA1002 strain lacks all the genes in the *pga* locus, and exhibits similar early biofilm architecture, it is essential to understand the disease causing potential of this strain to elucidate the role of PGA in *A*. *actinomycetemcomitans*-induced colonization and bone resorption in a rat model. Surprisingly, the results for the *pga* mutant strain showed that the bone resorption was indistinguishable from the non-inoculated control group (P = 0.43) both of which were significantly lower (P = 0.0006 and 0.0029) than the bone resorption observed for the wild-type fed group (Table 2; Fig. 4).

In other studies, the bone resorption induced by the *cdtB* mutant strain was comparable to the wild-type strain [14] while rats fed either *flp-1* or *tadA* mutant strains showed no bone resorption [13]. Thus, mutant strains lacking known virulent genes involved with colonization show reduced or no bone resorption. Interestingly, for the *pga* mutant fed rats in this study, an elevated antibody response was observed at 12 weeks (post-inoculation) (Table 2) which is comparable to the wild-type fed group. In spite of this elevated response, the bacterium is unable to induce bone resorption. However, the immune responses from the *flp-1* mutant fed rats were almost non-existent and indistinguishable from that of the control rat group [13]. In the *flp-1* mutant, where there is no colonization, the bone resorption result could be easily explained [13]. If there were no colonization, there would be no bone resorption. In the *pga* mutant, the observed results suggest that the role of PGA is not as straightforward as in the *flp-1* mutant. In a recent study, PGA has been proposed to play a role as a major fitness factor for bacterial survival. Using urinary track infection model, the authors showed that the wild-type strain outcompeted the *pga* mutant strain [32]. It is entirely possible that PGA in *A*. *actinomycetemcomitans* might also play such a role as a fitness factor and the lack of PGA production in EA1002 may have affected the colonization efficiency of the strain.

The gene expression data (Fig. 5) provide a further explanation for this attenuated colonization in our rat model. Because growth conditions can change the gene expression levels, it was necessary to maintain the growth conditions for wild-type and EA1002 to be identical. This would ensure that a proper comparison could be made between the two strains. As seen in Fig. 5, the absence of PGA production affect negatively on the mRNA expression levels of three important colonization mediating genes, *flp-1*, *aae* and *apiA*. Since there is about 2-fold lower *flp-1*, there would less colonization and could result in reduced or no bone resorption. It is also interesting to note that the expression levels of bacterial toxin genes involved in immune evasion were not affected by the absence of PGA. Given this data, we believe that in the early stages of biofilm growth (16 h biofilm), PGA production appears to be critical for survival of *A*. *actinomycetemcomitans* in the oral cavity and to induce bone resorption. Thus, our study has highlighted an additional role of PGA in *A*. *actinomycetemcomitans*-induced bone resorption. Clearly, the presence of PGA in *A*. *actinomycetemcomitans* is necessary for the expression of attachment genes, *flp-1*, *apiA* and *aae*, the two autotransporters involved in the process. A recent study has demonstrated that there is an enhanced expression of the complement resistance protein, ApiA, by sensing the metabolite hydrogen peroxide resulting in reduced killing of *A*. *actinomycetemcomitans* [33]. Our study showing that the reduction in the levels of *katA* and *apiA* is supportive of this study and further demonstrates the involvement of PGA in such resistance. Future studies elaborating the role of PGA by RNA-Seq analysis of the differential gene expression in the two strains is likely to provide a comprehensive list of genes that are affected in *A*. *actinomycetemcomitans* by the absence of PGA.

## Conclusions

The main conclusion that emerges from this study is that PGA plays an important role in the *A*. *actinomycetemcomitans*-induced bone resorption by modulating genes involved in the attachment stage during biofilm growth. Although the biofilm growth characteristics were similar between the two strains and the level of toxins was not affected, PGA appears to be required for the *A*. *actinomycetemcomitans* to be effective in tissue destruction. While previous studies highlighted the role of PGA in biofilm formation as well in immune evasion, our study demonstrates that PGA modulates the expression levels of virulence genes in the early stages of growth and it facilitates the understanding of the initial colonization process.
